# Supplementary material for: Splice-Junction-Based Mapping of Alternative Isoforms in the Human Proteome
Source: Cell Rep. Author manuscript; Available in PMC 2020 Jan 15. (PMC6961840; doi:10.1016/j.celrep.2019.11.026)
Supplement: 2 [file NIHMS1546469-supplement-2.zip › DF1/PXD010154/Heart/Heart_15_MYBPC3_TDSHEDTGILDFSSLLK.pdf]

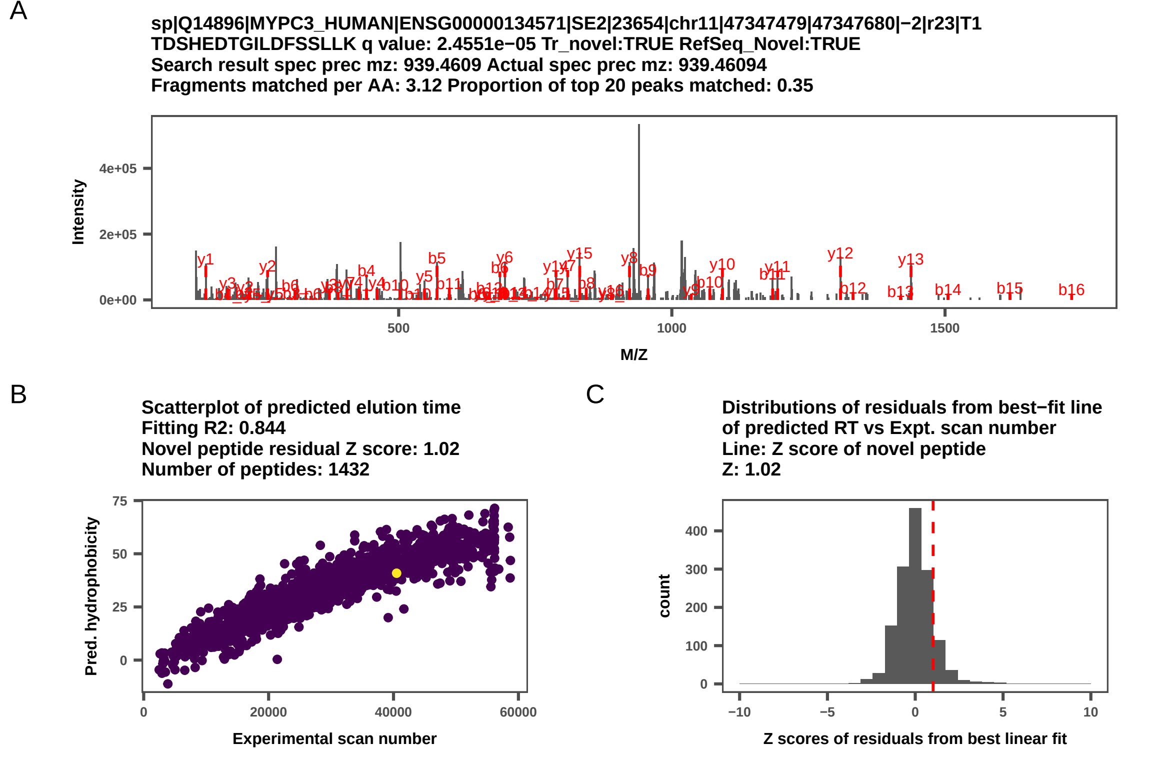
A
sp|Q14896|MYPC3_HUMAN|ENSG00000134571|SE2|23654|chr11|47347479|47347680|−2|r23|T1
TDSHEDTGILDFSSLLK q value: 2.4551e−05 Tr_novel:TRUE RefSeq_Novel:TRUE
Search result spec prec mz: 939.4609 Actual spec prec mz: 939.46094
Fragments matched per AA: 3.12 Proportion of top 20 peaks matched: 0.35
Intensity
4e+05
2e+05
0e+00
y1
y2
b4
b5
y5
y6
b6
b11
y15
y8
b9
y10
b10
y11
b11
y12
b12
y13
b13
b14
b15
b16
500
1000
1500
M/Z
B
Scatterplot of predicted elution time
Fitting R2: 0.844
Novel peptide residual Z score: 1.02
Number of peptides: 1432
Pred. hydrophobicity
75
50
25
0
0
20000
40000
60000
Experimental scan number
C
Distributions of residuals from best−fit line
of predicted RT vs Expt. scan number
Line: Z score of novel peptide
Z: 1.02
count
400
300
200
100
0
−10
−5
0
5
10
Z scores of residuals from best linear fit
